# Identification of symbiotic bacteria in the midgut of the medically important mosquito, *Culiseta longiareolata* (Diptera: Culicidae)

**DOI:** 10.1186/s13104-020-05220-0

**Published:** 2020-08-10

**Authors:** Fereshteh Ghahvechi Khaligh, Mozaffar Vahedi, Ali Reza Chavshin

**Affiliations:** 1 Department of Medical Entomology and Vector Control, School of Public Health, Urmia University of Medical Sciences, Urmia, Iran; 2 Social Determinants of Health Research Center, Urmia University of Medical Sciences, Urmia, Iran

**Keywords:** 16S rRNA, Mosquitoes, Symbionts, Paratransgenesis

## Abstract

**Objective:**

The potential use of symbiotic bacteria for the control of mosquito-borne diseases has attracted the attention of scientists over the past few years. *Culiseta longiareolata* is among the medically important mosquitoes that transmit a wide range of vector-borne diseases worldwide. However, no extensive studies have been done on the identification of its symbiotic bacteria. Given the role of this species in the transmission of some important diseases and its widespread presence in different parts of the world, including northwestern parts and the West Azerbaijan Province in Iran, a knowledge about the symbiotic bacteria of this species may provide a valuable tool for the biological control of this mosquito. Accordingly, the present study was conducted to isolate and identify the cultivable isolates bacterial symbionts of *Culiseta longiareolata* using 16S rRNA fragment analysis.

**Results:**

The midguts of 42 specimens of *Cs. longiareolata* were dissected, and the bacteria were cultured on agar plates. After the purification of the bacterial colonies, 16srRNA region amplification and gene sequence analysis were performed, and the sequences were confirmed by biochemical methods. In the present study, 21 isolates belonging to the genera *Acinetobacter*, *Aerococcus*, *Aeromonas*, *Bacillus*, *Carnobacterium*, *Klebsiella*, *Morganella*, *Pseudomonas*, *Shewanella* and *Staphylococcus* were identified.

## Introduction

Acting as vectors of diseases, mosquitoes transmit a wide range of parasite and arbovirus pathogens which are of veterinary and medical importance [1, 2]. Some species of mosquitoes are widely distributed throughout the world and are involved in the transmission cycle of a notable number of mosquito-borne diseases.

Among the veterinary and medically important mosquito species is the multivoltine *Culiseta longiareolata*. This species is thermophilic and highly ornithophilic [3]. It is widely distributed in Europe, Asia, Africa, and the Mediterranean Sea [4], and acts as the vector of some infectious diseases such as the avian malaria [5, 6], tularemia [3], and arboviruses like West Nile fever [7–9].

Since mosquito-borne diseases cause serious health problems in many parts of the world, identifying different aspects of the biology of mosquito is of great importance. Knowledge about the biological properties, environmental requirements, and food chains [10, 11] of mosquitoes can be utilized for biological control. The symbiotic microbiota associated with mosquitoes have been found to affect most of their biological activities [12–14].

The symbiotic microbiota associated with each mosquito and their role in the biological activities of the mosquitoes can provide a valuable tool for the biological control of disease vectors [15–21]. Symbiotic bacteria affect the development [22, 23], nutrition [24, 25], reproduction [26–28], defense mechanisms [29, 30], and immunity [31] of mosquitoes. To understand the effect of symbiotic bacteria in the biological control of mosquitoes or mosquito-borne diseases, accurate identification of the symbiotic bacteria associated with each vector is an important first step [32, 33].

Although symbiotic bacteria have been studied and identified in different mosquito species [33–42], so far, no study has been performed on the identification of bacterial symbionts of *Cs. longiareolata*.

Given the role of this species in the transmission of some important diseases and its widespread presence in different parts of the world, including the northwestern region [34, 43–46] and West Azerbaijan Province (which shares border with four countries) in Iran, the symbiotic bacteria of *Culiseta longiareolata* were investigated in this study. In the present study, the cultivable bacterial symbionts of *Culiseta longiareolata* were isolated, cultivated and identified using 16S rRNA fragment analysis.

## Main Text

### Material and Methods

#### Field collection of *Cs. longiareolata* and isolation of midgut bacteria

Mosquitoes specimens were collected from three regions of Urmia County (1- Naz-Loo: 37.651213, 44.983285, 2- Ghahraman-Loo: 37.659869, 45.207550, and 3- Moallem 37.546660, 45.033280) in the West Azerbaijan Province in the Northwestern region of Iran (Additional file 1: Figure S1) during May–August 2018 using different previously described collection methods [47]. The collection techniques used in this study included the standard dipping method for larvae collection, and hand catches, day and night landing catches on cows, total catch, and pit shelter collection for adult specimens. The specimens were transferred alive to the entomology laboratory of the Department of Medical Entomology in the School of Public Health, and species were identified using morphological characteristics-based keys [48].

Adult female specimens of *Cs. Longiareolata* were identified and used for gut bacteria isolation. These specimens were sterilized, and their midguts were dissected individually under sterile conditions, according to previously described methods [33, 39].

The dissected midguts were mashed and suspended in 500 μL of Brain Heart Infusion (BHI), and the suspension was incubated at 28 ± 2 °C and 200 rpm for 24 h. A 100 μL aliquot of the midgut contents was serially diluted up to 10^−6^ and plated onto Nutrient Agar (Merck, Germany) and incubated at 28 ± 2 °C for 24–48 h [39]. Continuous sub-culture of each bacterial colony using the streaking method was done to isolate single purified colonies of the bacteria. The individual colonies of the bacteria were later used for DNA extraction and PCR, biochemical and phenotyping studies.

#### 16S rRNA gene amplification and sequencing

All purified bacterial colonies were individually subjected to genomic DNA extraction using the FavorPrep™ Kit (Favorgen, Taiwan), according to the manufacturer’s instructions. The 16S rRNA universal primers and previously described PCR program were used to amplify the 16S rRNA fragment [49]. The acquired PCR amplicons were sequenced by Microsynth (Swiss).

All acquired sequences were checked for the presence of probable chimeric sequences by the Mallard program (https://www.bioinformatics-toolkit.org). All suspicious sequences were removed from the data set, and the resulting sequences were analyzed. The sequences were compared to the databases of the Ribosomal Database Project (RDP II; Michigan State University: rdp.cme.msu.edu) and the GenBank (www.ncbi.nlm.nih.gov/BLAST). Isolates were identified at the Genus and Species level based on sequence comparison using the GenBank and RDPII entries.

Finally, sequencing results that were consistent with the results of the biochemical studies were considered as reliable and definitive sequence of the bacterial isolates.

The MEGA7 [50] was used for phylogenetic analysis and tree construction. The Maximum Likelihood (ML) method was used for the phylogenetic tree construction based on the Tamura 3-parameter model [51] (1000 bootstrap replicates) analyses.

### Results

In the present study, five species belonging to three genera of mosquitoes were collected and identified (*An. maculipennis, Culex modestus, Cx. pipiens, Cx. theileri *and* Cs. longiareolata*) in three sites across the Urmia County.

After species identification, specimens of *Cs. longiareolata* were selected for the purpose of the study. The midguts of 42 specimens of *Cs. longiareolata* were dissected, and the bacteria were cultured on agar plates to obtain bacterial colonies. After the purification of the bacterial colonies, 16srRNA region amplification and gene sequence analysis were performed for the bacterial isolates, and the sequences were confirmed by biochemical methods. In the present study, 21 isolates belonging to ten genera of bacteria were identified. The bacteria genera identified in this study include, *Acinetobacter*, *Aerococcus*, *Aeromonas*, *Bacillus*, *Carnobacterium*, *Klebsiella*, *Morganella*, *Pseudomonas*, *Shewanella,* and *Staphylococcus*. All acquired sequences were deposited in GenBank. The accession nos. of the bacterial species have been presented in Table 1.

**Table 1:** Bacteria of midgut of *Cs. longiareolata* and their accession numbers

Genus	Species/isolate	Accession No	Gram’s staining
*Acinetobacter*	*radioresistens Urmia-Culis-b*	MK840759	N
*Aerococcus*	*urinaeequi Urmia-Culis-12*	MK840745	P
*Aeromonas*	*hydrophila Urmia-Culis-6*	MK840743	N
	*salmonicida Urmia-Culis-13*	MK840746	N
*Bacillus*	*safensis Urmia-Culis-18*	MK840747	P
	*safensis Urmia-Culis-20*	MK840748	P
	*sp. Urmia-Culis-48*	MK840755	P
	*subtilis Urmia-Culis-49*	MK840756	P
	*sp. Urmia-Culis-50*	MK840757	P
	*pumilus Urmia-Culis-63*	MK840758	P
	*safensis Urmia-Culis-f*	MK840760	P
	*sp. Urmia-Culis-g*	MK840761	P
*Carnobacterium*	*maltaromaticum Urmia-Culis-11*	MK840744	P
*Klebsiella*	*oxytoca Urmia-Culis-34*	MK840752	N
	*oxytoca Urmia-Culis-46*	MK840754	N
*Morganella*	*morganii Urmia-Culis-29*	MK840749	N
	*morganii Urmia-Culis-32*	MK840751	N
*Pseudomonas*	*protegens Urmia-Culis-30*	MK840750	N
	*sp. Urmia-Culis-3*	MK840741	N
*Shewanella*	*sp. Urmia-Culis-4*	MK840742	N
*Staphylococcus*	*epidermidis Urmia-Culis-36*	MK840753	P

Among the ten identified bacteria Genera, six were Gram-negative (*Acinetobacter, Aeromonas, Klebsiella*, *Morganella*,* Pseudomonas* and *Shewanella*) and four Genera were Gram-positive (*Aerococcus*, *Bacillus*, *Carnobacterium* and *Staphylococcus*).

Among the 21 isolates from the midgut of adult *Cs. longiareolata*, *Aeromonas* was the most frequent symbiont with eight isolates. Two species belonging to each of the genera *Aeromonas*, *Klebsiella*, *Morganella*, and *Pseudomonas* were also isolated and identified from the midgut of adult *Cs. longiareolata*.

**Fig. 1:**
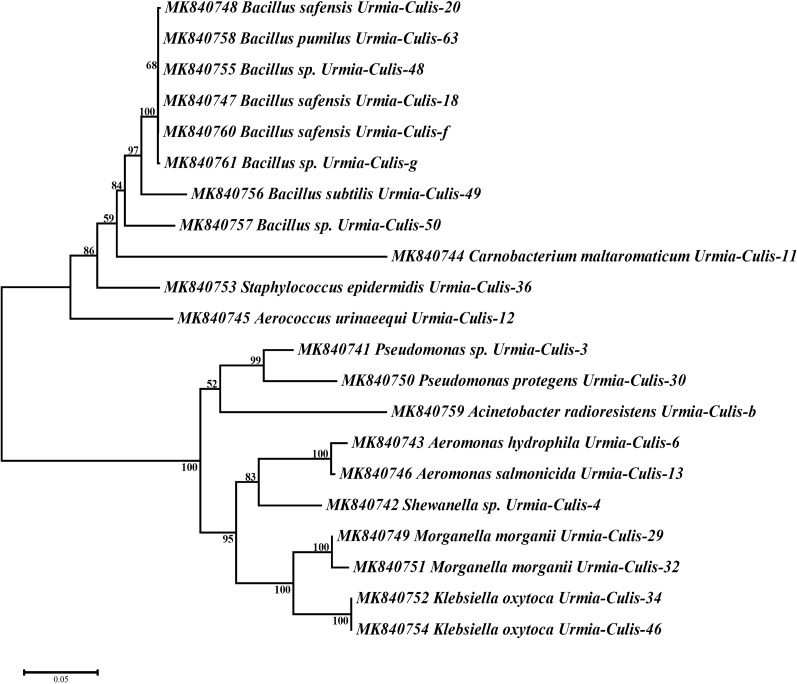
Evolutionary relationships of bacterial symbionts of *Cs. longiareolata. *The evolutionary history was inferred using the Neighbor-Joining method [60]. The optimal tree with the sum of branch length = 1.08886518 is shown. The percentage of replicate trees in which the associated taxa clustered together in the bootstrap test (1000 replicates) are shown next to the branches [61]. The tree is drawn to scale, with branch lengths in the same units as those of the evolutionary distances used to infer the phylogenetic tree. The evolutionary distances were computed using the Maximum Composite Likelihood method [62] and are in the units of the number of base substitutions per site. The analysis involved 21 nucleotide sequences. All positions containing gaps and missing data were eliminated. There were a total of 879 positions in the final dataset. Evolutionary analyses were conducted in MEGA7 [50]

Interestingly, the phylogenetic analysis of the acquired sequences of the bacteria isolates showed distinct monophyletic clades based on gram staining properties of their cell wall (Gram-negative and Gram-positive bacteria) (Fig. 1).

**Fig. 2:**
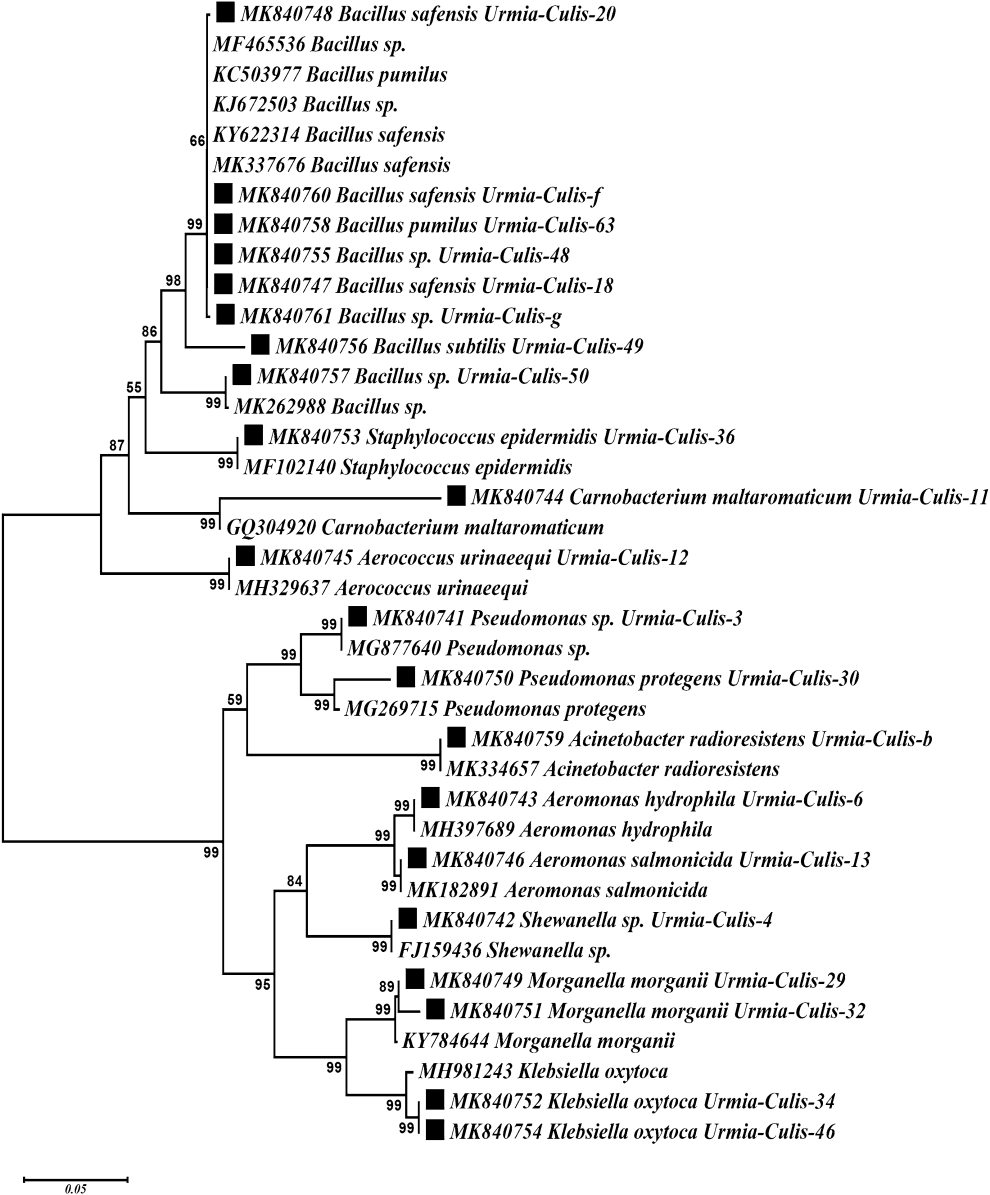
Evolutionary relationships of bacterial symbionts of *Cs. longiareolata* (indicated by ■), compared with other sequences retrieved from GenBank). The evolutionary history was inferred using the Neighbor-Joining method [60]. The optimal tree with the sum of branch length = 0.96545507 is shown. The percentage of replicate trees in which the associated taxa clustered together in the bootstrap test (1000 replicates) are shown next to the branches [61]. The tree is drawn to scale, with branch lengths in the same units as those of the evolutionary distances used to infer the phylogenetic tree. The evolutionary distances were computed using the Maximum Composite Likelihood method [62] and are in the units of the number of base substitutions per site. The analysis involved 38 nucleotide sequences. Codon positions included were 1st + 2nd + 3rd. All positions containing gaps and missing data were eliminated. There were a total of 876 positions in the final dataset. Evolutionary analyses were conducted in MEGA7 [50]

Also, phylogenetic analysis of the sequences obtained from the present study and similar sequences retrieved from the GenBank revealed the placement of bacteria of the same species and Genera in common branch and clades (Fig. 2).

### Discussion

The present study is the first report on the bacterial symbionts associated with the midgut of *Cs. longiareolata.* This mosquito vector plays a notable role in the transmission and maintenance of the transmission cycle of important diseases such as avian malaria [5, 6], tularemia [3], and arboviruses like West Nile fever [7–9] as secondary a vector.

The results of the midgut symbiotic bacteria of this vector are consistent with the results of many studies conducted on other vectors. In previous studies, symbiotic bacteria isolated from the midgut of *Aedes aegypti* [52] and *Cx. quinquefasciatus* [53] were predominantly members of the genus *Bacillus, Klebsiella, Pseudomonas* and *Staphylococcus, which is consistent with the results of the present study.* In another study conducted in India, members of the Genus *Aeromonas* were isolated from *Cx. quinquefasciatus* [54], which is also in agreement with the present study.

Symbiotic bacteria belonging to the genera *Morganella*, *Aeromonas*, and *Klebsiella* have also been identified in *Anopheles fluviatilis* [55], which is similar to the findings of our study.

Concerning the result of the present study, which identified the predominant isolates in the midgut of *Cs. longiareolata*, this finding is in agreement with the results of the dominant bacteria in the midgut of *An. stephensi* and *An. culicifacies* [33, 39], *Aedes aegypti* [56].

The identification of suitable candidates for paratransgenesis in the use of symbionts for biological control of vectors is of major interest to researchers. Members of the Genus *Pseudomonas* have been suggested in some studies as suitable candidates for paratransgenesis [16, 32, 35, 57–59]. In the present study, members of the genus *Pseudomonas* were identified in *Cs. longiareolata,* which confirms the results of previous studies which have reported the wide range of presence of *Pseudomonas* bacteria in different mosquito species.

In the first part of the study, different mosquito species were collected and identified. We captured five species of mosquitoes (*An. maculipennis, Culex modestus, Cx. pipiens, Cx. theileri* and *Cs. longiareolata*) in the study area. Previous studies have also identified these mosquito species in the northwest of Iran [43–46].

The five species captured in this study are important vectors of human and animal diseases. The geographical location of the northwest region of Iran (shares border with four countries) and the climatic diversity, as well as the history of mosquito-borne diseases makes this region vulnerable to a wide variety of mosquitoes. The presence of these vectors in this region requires public health attention, and the design of appropriate control programs is necessary to prevent the occurrence of epidemics.

### Conclusion

The present study identified bacterial symbionts of *Cs. longiareolata*. To the best of our knowledge, this is the first report of bacteria symbiont of *Cs. longiareolata*. Is is recommended that future research in this area focus more precisely on identifying the biological properties of the isolated symbiotic bacteria, their biodiversity, and the biological relationship with their hosts, with the aim of developing new symbiont-based control programs. Previous studies have suggested that members of the Genus *Pseudomonas may be* suitable candidates for paratransgenesis. The isolation of *Pseudomonas spp.* in the present study confirms the wide spread of this genus in mosquito species and may further support the use of this species as a candidate for paratransgenesis to control mosquito-borne diseases.

## Limitations

Only the symbionts of the adult stage of *Cs. longiareolata* were identified.

## Supplementary information

**Additional file 1: Figure S1.** Location of West Azerbaijan Province and Urmia County and sampling localities, 1—Naz-Loo: 37.651213, 44.983285, 2—Ghahraman-Loo: 37.659869, 45.207550, and 3—Moallem: 37.546660, 45.033280 (Original basic map has been prepared from d-maps.com).

## Data Availability

The datasets used and/or analysed during the current study are available from the corresponding author on reasonable request.

## Abbreviations

*Cs. longiareolata*: *Culiseta longiareolata*
16S rRNA: 16 s ribosomal `RNA
