# Supplementary figures and images for: Identification of symbiotic bacteria in the midgut of the medically important mosquito, Culiseta longiareolata (Diptera: Culicidae)
Source: BMC Res Notes. 2020 Aug 10;13:378. doi: 10.1186/s13104-020-05220-0 (PMC7418411; doi:10.1186/s13104-020-05220-0)

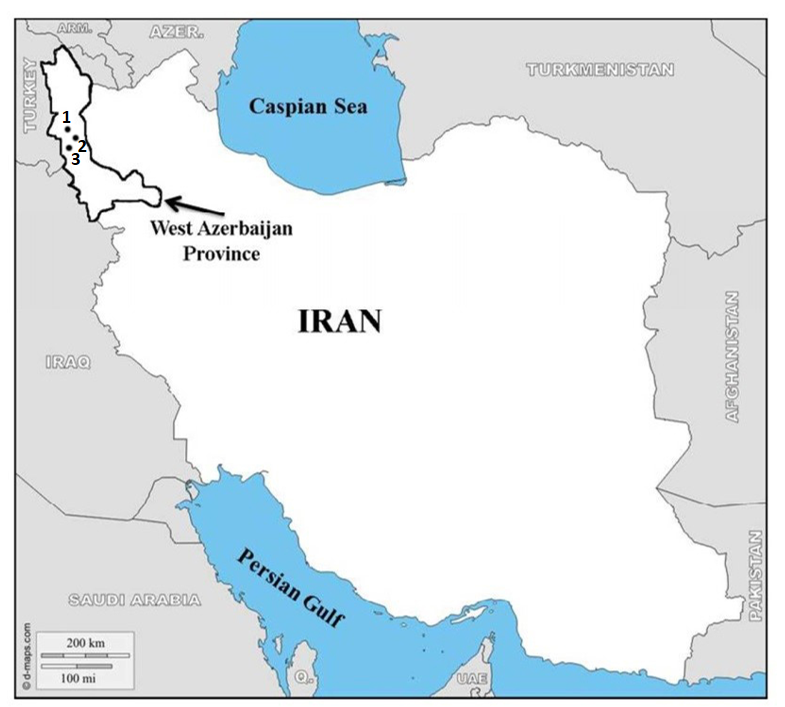

Supplement: Supplementary file 1 — Additional file 1: Figure S1. Location of West Azerbaijan Province and Urmia County and sampling localities, 1—Naz-Loo: 37.651213, 44.983285, 2—Ghahraman-Loo: 37.659869, 45.207550, and 3—Moallem: 37.546660, 45.033280 (Original basic map has been prepared from d-maps.com). [file 13104_2020_5220_MOESM1_ESM.tif]
